# Structural insights into the methyl donor recognition model of a novel membrane-binding protein UbiG

**DOI:** 10.1038/srep23147

**Published:** 2016-03-15

**Authors:** Yuwei Zhu, Xuguang Jiang, Chongyuan Wang, Yang Liu, Xiaojiao Fan, Linjuan Zhang, Liwen Niu, Maikun Teng, Xu Li

**Affiliations:** 1 Hefei National Laboratory for Physical Sciences at Microscale, Innovation Center for Cell Signaling Network, School of Life Science, University of Science and Technology of China, Hefei, Anhui, 230026, People’s Republic of China; 2 Key Laboratory of Structural Biology, Hefei Science Center of CAS, Chinese Academy of Science, Hefei, Anhui, 230026, People’s Republic of China

## Abstract

UbiG is a SAM-dependent *O*-methyltransferase, catalyzing two *O*-methyl transfer steps for ubiquinone biosynthesis in *Escherichia coli*. UbiG possesses a unique sequence insertion between β4 and α10, which is used for membrane lipid interaction. Interestingly, this sequence insertion also covers the methyl donor binding pocket. Thus, the relationship between membrane binding and entrance of the methyl donor of UbiG during the *O*-methyl transfer process is a question that deserves further exploration. In this study, we reveal that the membrane-binding region of UbiG gates the entrance of methyl donor. When bound with liposome, UbiG displays an enhanced binding ability toward the methyl donor product S-adenosylhomocysteine. We further employ protein engineering strategies to design UbiG mutants by truncating the membrane interacting region or making it more flexible. The ITC results show that the binding affinity of these mutants to SAH increases significantly compared with that of the wild-type UbiG. Moreover, we determine the structure of UbiG∆^165–187^ in complex with SAH. Collectively, our results provide a new angle to cognize the relationship between membrane binding and entrance of the methyl donor of UbiG, which is of benefit for better understanding the *O*-methyl transfer process for ubiquinone biosynthesis.

Ubiquinone (coenzyme Q), an essential lipid in the electron transport chain, is found in the inner mitochondrial membrane of eukaryotes as well as the plasma membrane of prokaryotes^1^,^2^. Ubiquinone plays a pivotal role in shuttling electrons from complex I or II to complex III for ATP synthesis in bacteria and higher eukaryotes^3^. In *Homo sapiens*, ubiquinone is tightly related to a number of diseases like muscular, cancer, diabetes and neurodegenerative disorders^4^,^5^,^6^. The biosynthesis of ubiquinone between prokaryotes and eukaryotes is similar, both beginning with the assembly of a quinone head group and a variable-length hydrophobic isoprenoid tail. Then, modifications of the benzoquinone are followed, including *C*-hydroxylation, decarboxylation, *O*-methylation and *C*-methylation^7^,^8^,^9^.

UbiG, a 240-residues protein in *E. coli*, is identified to be essential for ubiquinone biosynthesis *in vivo*. Mutations in the *ubiG* gene could cause ubiquinone deficiency^10^. UbiG belongs to the Class I SAM-dependent-methyltransferases family, catalyzing the transfer of the methyl group from SAM to substrate^11^,^12^. In *E. coli*, the biosynthesis of ubiquinone needs two *O*-methylation steps, both of which are catalyzed by UbiG. The first *O*-methylation step is converting 2-polyprenyl-6-hydroxyphenol to 2-polyprenyl-6-methoxyphenol. The second step involves the *O*-methylation of 2-polyprenyl-3-methyl-5-hydroxy-6-methoxy-1,4-benzoquinol to form ubiquinone^13^.

Notably, unlike other types of methylation processes, the *O*-methylation reaction for ubiquinone biosynthesis *in vivo* is membrane associated^14^. The structure of full-length UbiG was determined and analyzed by our previous studies^15^. UbiG exhibits a globular fold, and the core structure comprises eight-stranded β sheet. Compared with the typical Class I SAM-dependent *O*-methyltransferases, UbiG possesses a unique sequence insertion shaping a membrane interaction patch. Meanwhile, our previous work indicated that UbiG binds preferentially to phosphatidylglycerol (PG) and cardiolipin (CL), two major components of *E. coli* plasma membrane, and the mutation compromising UbiG membrane interaction largely diminishes the growth rate of *E. coli* cells, revealing that the membrane-binding ability is pivotal for the function of UbiG *in vivo*^15^. Nevertheless, due to the lack of further structural information, the methyl donor recognition model of UbiG remains unclear. Furthermore, the significance of the membrane-binding ability of UbiG in the *O*-methyl transfer process for ubiquinone biosynthesis is still worth exploring.

Here, we construct an UbiG mutant (UbiG∆^165–187^) by deleting the sequence insertion that covers the methyl donor binding pocket. The binding affinity of UbiG∆^165–187^ to SAH is approximately 58-fold higher than that of wild-type UbiG. Moreover, both wild-type UbiG bound to liposome and UbiG mutants that weaken the interaction of this sequence insertion with the core component show an enhanced binding ability toward SAH. Finally, we solve the crystal structure of UbiG∆^165–187^ complexed with SAH at 2.10 Å. Taken together, our results uncover the methyl donor diffusion mechanism of UbiG, and reveal that the membrane association of UbiG may regulate the entrance of methyl donor, which suggests an inextricable relationship between membrane anchoring and *O*-methyl transfer reaction in the ubiquinone biosynthesis pathway.

## Results and Discussion

### UbiG bound with liposome displays an enhanced binding ability toward SAH

**Figure 1:**
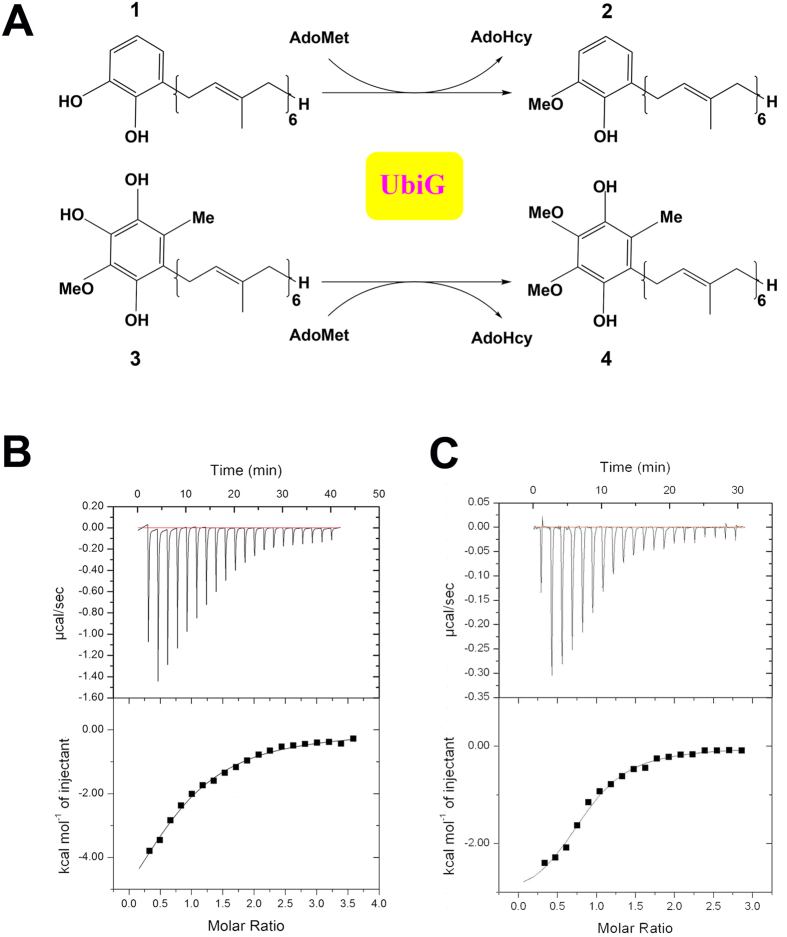
Membrane association promotes UbiG interacting with SAH. *O*-Methyltransferase reactions catalysed by UbiG in ubiquinone biosynthesis. (**A**) UbiG catalyzes two *O*-Methyltransferase steps in ubiquinone biosynthesis. The first *O*-methylation step is converting 2-polyprenyl-6-hydroxyphenol (*compound 1*) to 2-polyprenyl-6-methoxyphenol (*compound 2*). The second step involves the *O*-methylation of 2-polyprenyl-3-methyl-5-hydroxy-6-methoxy-1,4-benzoquinol (*compound 3*) to form ubiquinone (*compound 4*). ITC profile of SAH titrated against wild-type UbiG (**B**) and liposome-bound UbiG (**C**). The upper panels showed the raw ITC data for injection of ligands into the sample cell containing wild-type UbiG or liposome-bound UbiG. The peaks were normalized to the ligand: protein molar ratio, and were integrated as shown in the bottom panels. Solid dots indicated the experimental data, and their best fit was obtained from a nonlinear least squares method, using a one-site binding model depicted by a continuous line.

Our previous results have reported the crystal structure of UbiG from *E. coli*, and identified the residues vital for membrane binding. Interestingly, these residues mainly locate in helix α9 and loop α9/α10, a region that covers the possible methyl donor binding pocket^15^. Moreover, to gain insight into the methyl donor recognition model of UbiG, we tried to determine the complex structure of UbiG with SAH. However, we failed to obtain the complex structure by either co-crystallization or crystal soaking. To investigate whether the membrane association of UbiG influences the diffusion of methyl donor, we compare the binding affinity of wild-type UbiG and liposome-bound UbiG to SAH (Table 2). The ITC experiments show that wild-type UbiG bound SAH with a *K*_d_ of 104.43 ± 17.21 μM (Fig. 1B), whereas the affinity of liposome-bound UbiG to SAH (*K*_d_ = 9.63 ± 2.10 μM) increased ≈11-fold (Fig. 1C), indicating that the membrane association promotes UbiG interacting with SAH.

### The membrane binding region of UbiG gates the entrance of methyl donor

**Figure 2:**
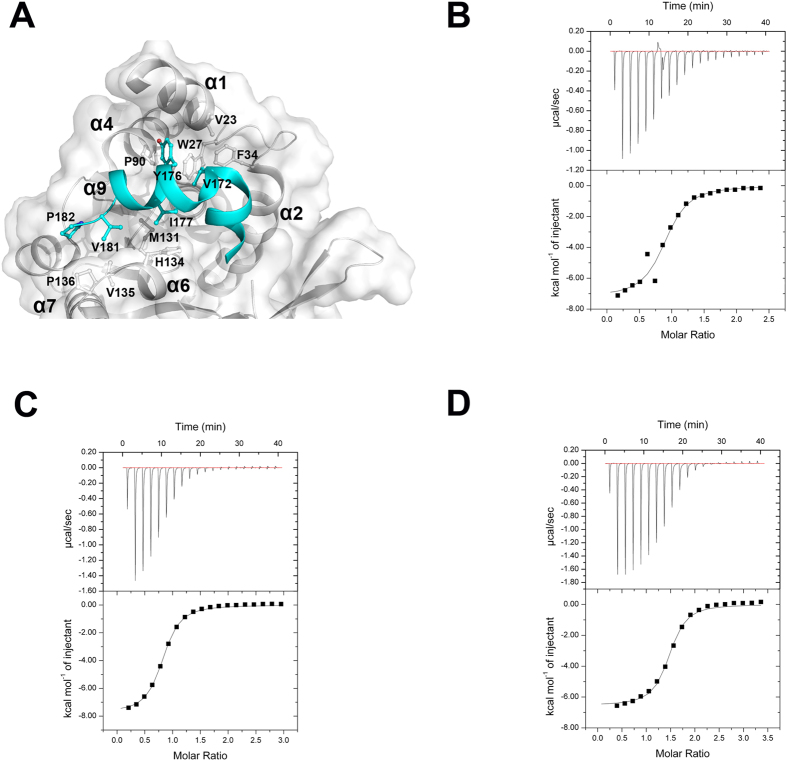
The membrane binding region of UbiG gates the entrance of methyl donor. (**A**) Surface show of the structure of UbiG. The insertions of structural elements in UbiG are colored cyan. Residues involved in the hydrophobic interaction network of α9 with the core structure of UbiG are labelled. ITC profile of SAH titrated against UbiG-M1 (**B**), UbiG-M2 (**C**) and UbiG∆^165–187^ (**D**).

In the structure of UbiG, the membrane binding region including α9 and loop α9/α10 is stabilized by hydrophobic interactions with the core structure. As shown in Fig. 2(A), residues Val^172^ (helix α9), Tyr^176^ (helix α9), Ile^177^ (helix α9), Val^181^ (loop α9/α10) and Pro^182^ (loop α9/α10) make extensive hydrophobic contacts with residues Val^23^ (helix α1), Trp^27^ (loop α1/α2), Phe^34^ (helix α2), Pro^90^ (helix α4), Met^131^(helix α6), His^134^ (helix α6), Val^135^ (helix α6), and Pro^136^ (loop α6/α7) of the core structure of UbiG. To investigate whether this membrane binding region affects the diffusion of methyl donor, we construct UbiG mutants to enhance the flexibility of this region. We designed two UbiG mutants, UbiG-M1 (residues Val^172^ and Tyr^176^ mutated to Ala) and UbiG-M2 (residues Ile^177^, Val^181^ and Pro^182^ mutated to Ala) to weaken the interaction of this membrane binding region with the core structure of UbiG. The ITC experiments show that the binding affinity of UbiG-M1 to SAH was 3.37 ± 0.84 μM, increasing ≈31-fold compared with that of wild-type UbiG (Fig. 2B). UbiG-M2 bound SAH with a *K*_d_ of 2.77 ± 0.36 μM, increasing ≈38-fold compared with that of wild-type UbiG (Fig. 2C). To further confirm our hypothesis, we designed another UbiG mutant (UbiG∆^165–187^) by deleting this membrane interacting region that covers the methyl donor binding pocket. We compared the binding affinity of wild-type UbiG and UbiG∆^165–187^ to SAH by ITC experiments. The binding affinity of UbiG∆^165–187^ to SAH was 1.84 ± 0.16 μM, increasing ≈57-fold compared with that of wild-type UbiG (Fig. 2D). These data strongly support our hypothesis, and confirm that in the membrane-unbound state, the membrane binding region of UbiG hinds the entrance of methyl donor.

### Structure of UbiG∆^165–187^ in complex with SAH

**Figure 3:**
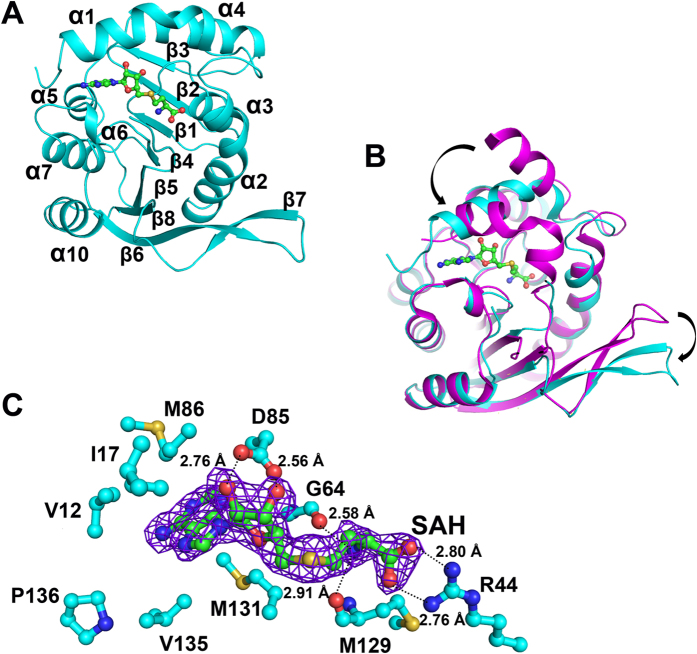
Structure of UbiG∆^165–187^ in complex with SAH. (**A**) Cartoon show of the overall structure of UbiG∆^165–187^ in complex with SAH. The α-helices and β strands are labelled and colored cyan. The methyl donor product SAH is shown as a ball-and-stick model and is colored green. (**B**) Superimposition of the structures of wild-type UbiG and UbiG∆^165–187^ in complex with SAH. Wild-type UbiG and UbiG∆^165–187^ are colored magenta and cyan, respectively. (**C**) SAH binding model of UbiG∆^165–187^. The 2Fo-Fc electron density map (contoured at 1σ) for SAH is shown as blue. The residues involved in interacting with SAH of UbiG∆^165–187^ are labelled and colored cyan. The dashed lines represent hydrogen bonds.

To disclose the accurate recognition pattern of SAH, we crystallized UbiG∆^165–187^ in complex with SAH at a resolution of 2.10 Å. The details of the data collection and refinement statistics are summarized in Table 1. The final model contains one molecule of UbiG∆^165–187^ and one molecule of SAH, with a stoichiometry of 1:1. Due to the insufficient electron density, the N-terminal 9 residues could not be traced. UbiG∆^165–187^ displays a similar fold as wild-type UbiG (Fig. 3A). The overall main-chain root-mean squared deviation (RMSD) between UbiG∆^165–187^ and wild-type UbiG is 0.397 Å for 215 comparable Cα atoms. Comparison with the structure of wild-type UbiG, helix α1 of UbiG∆^165–187^ moves toward the SAH binding pocket and forms extensive hydrophobic interactions with the carbon-skeleton of SAH (Fig. 3B). In addition, due to the lack of the hydrophobic packing with helix α8, the β6 and β7 of UbiG∆^165–187^ move away from the core structure (Fig. 3B).

The electron density for the SAH is well defined in the final model of UbiG∆^165–187^ and the SAH is bound via an extensive hydrogen bond network and hydrophobic interaction. In light of the structure, we easily identify the SAH binding sites. The adenine ring of SAH is located in a hydrophobic pocket constituted by residues Val^12^, Ile^17^, Met^86^, Met^131^, Val^135^, and Pro^136^ (Fig. 3C). The ribosyl moiety is anchored via hydrogen bonds from the O2′ and O3′ hydroxyl groups to the side chain of Asp^85^ (Fig. 3C). The SAH carboxyl is locked by the side chain of Arg^44^, whereas the corresponding SAH amine is anchored to the main chain carbonyl oxygen atoms of Gly^64^ and Met^129^ via hydrogen bonds (Fig. 3C).

### The methyl donor binding model and diffusion mechanism of UbiG

**Figure 4:**
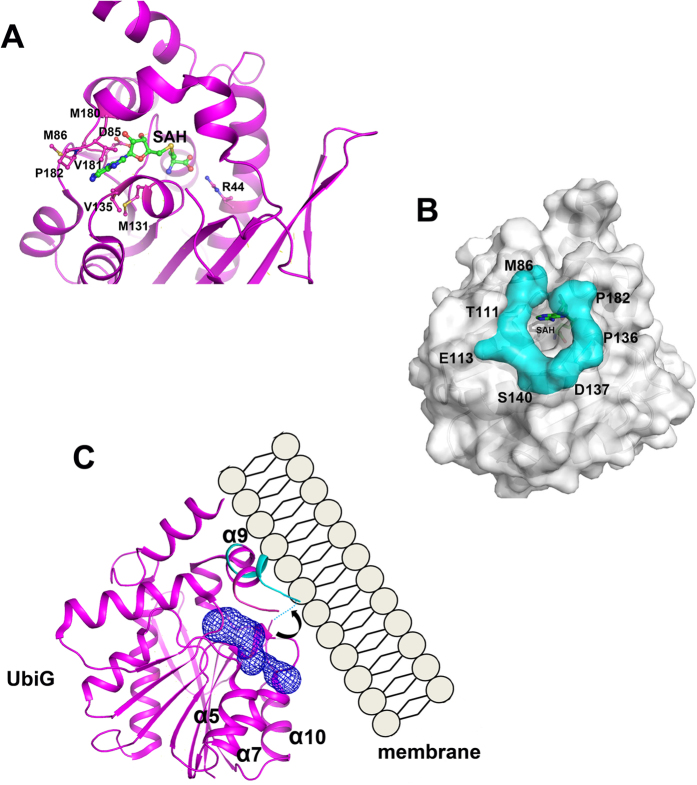
The methyl donor binding model and diffusion mechanism of UbiG. (**A**) A proposed SAH recognition model of UbiG. Residues involved in the interaction with SAH are labelled. (**B**) Surface show of the structure of UbiG. The residues gated the diffusion of the methyl donor are labelled and colored cyan. (**C**) Cartoon representation of the methyl donor diffusion mechanism of UbiG. Putative SAH access tunnel is calculated by *CAVER* and is denoted in mesh (blue). When UbiG associates with the membrane, strong hydrophobic driving forces may loosen the interaction of this membrane binding region with the core structure, and cause a relatively open channel for the diffusion of methyl donor during the O-methyl transfer process for ubiquinone biosynthesis.

Superimposition of the structures of wild-type UbiG and UbiG∆^165–187^ in complex with SAH, we map the SAH binding model of wild-type UbiG. As shown in Fig. 4(A), SAH is situated in the central cavity of the Rossmann-fold domain of UbiG. The interaction between UbiG and SAH can be divided into three parts in accordance to the moieties of SAH. For the adenine moiety, hydrophobic residues Met^86^, Met^131^, Val^135^, Met^180^, Val^181^ and Pro^182^ make extensive van der Waals interactions with the adenine ring (Fig. 4A). For the ribosyl moiety, the side chain of Asp^85^ forms two hydrogen bonds with the O2′ and O3′ hydroxyl groups (Fig. 4A). The interaction between UbiG and the homocysteine moiety of SAH is dominated by four hydrogen bonds. The side-chain of Arg^44^ contributes to two hydrogen bonds with the amino group of the homocysteine (Fig. 4A). The carboxyl group of the homocysteine makes another two hydrogen bonds with the main-chain carbonyl oxygen atoms of Gly^64^ and Met^129^, respectively (Fig. 4A). Then, we used the program *CAVER* to analyse the diffusion pathway of the methyl donor, which revealed a tunnel gated by residues Met^86^, Thr^111^, Glu^113^, Pro^136^, Asp^137^, Ser^140^, and Pro^182^ (Fig. 4B). This gate seems much narrow compared with that of most other class I SAM-MTases, such as catechol *O*-methyltransferase COMT (PDB code 1VID)^16^, rebeccamycin sugar 4′-*O*-Methyltransferase RebM (PDB code 3BUS)^17^, and 2-methoxy-6-polyprenyl-1,4-benzoquinone 5′-*C*-methyltransferase Coq5 (PDB code 4OBX)^18^, in which the methyl donor binding pocket is uncovered.

Combining with the ITC results mentioned above, we conclude that in the membrane-unbound state, the diffusion of methyl donor of UbiG is greatly affected by the narrow gate constituted by the membrane binding region. When UbiG associates with the membrane, strong hydrophobic driving forces may loosen the interaction of this membrane binding region with the core structure, and cause a relatively open channel for the diffusion of methyl donor during the *O*-methyl transfer process for ubiquinone biosynthesis (Fig. 4C). Association of membrane-bound proteins with the surface of cellular membranes usually plays a necessary role for a large variety of cellular functions. For example, the cytoskeleton uses the lipid-binding domain for directly anchoring to the membrane surface^19^. Bin-Amphiphysin-Rvs (BAR) domain containing proteins bind to the membrane surface to act as membrane shapers^20^. The attaching of alpha-toxin to membrane surface pushes the opening of the active center, which is help for hydrolysis of membrane phospholipids^21^,^22^. As we known, the *O*-methyl transfer reaction for ubiquinone biosynthesis catalyzed by UbiG is membrane associate *in vivo*^14^. Obviously, the membrane anchoring ability of UbiG is of benefit for sequestering substrates located in the lipid bilayer. In this study, we find surprisingly that the membrane association of UbiG also regulates the entrance of methyl donor, thus activating the *O*-methyl transfer reaction for ubiquinone biosynthesis. Our results provide much insight into the role of membrane association in regulating the enzyme activity of UbiG, and enhance our better understanding of the *O*-methyl transfer process for ubiquinone biosynthesis *in vivo*.

## Materials and Methods

### Cloning, expression and purification

Full-length UbiG from *E. coli* was expressed and purified as described previously^23^. UbiG mutants was generated by PCR with the MutanBEST Kit (TaKaRa) using the parent expression plasmid pET28a-UbiG (1-240) as template. The mutant plasmids were confirmed by DNA sequencing (Invitrogen). Plasmids containing the confirmed UbiG mutations were then transformed into *E. coli* BL21 (DE3) strain (Novagen), and the corresponding overproduced recombinant mutant proteins were purified as described for the wild-type UbiG^23^.

### Crystallization, data collection and processing

Crystallization trials were conducted using the hanging drop vapour diffusion method at 287 K. The protein UbiG∆^165–187^ was concentrated to approximately 16 mg/ml. The UbiG∆^165–187^-SAH complex was prepared by mixing UbiG∆^165–187^ with SAH at a 1:3 molar ratio. Diffraction quality crystals of UbiG∆^165–187^-SAH complex were obtained with 0.1 M citric acid pH 5.0 and 20% (v/v) 2-Methyl-2,4-pentanediol. For data collection, the crystals were cryo-protected using 25% (v/v) glycerol supplemented with crystallization solution, and flashed cool in liquid nitrogen. Diffraction data sets for the UbiG∆^165–187^-SAH complex were collected on beamline 19U of the Shanghai Synchrotron Radiation Facility (SSRF) using a CCD detector. All frames were collected at 100 K using a 1° oscillation angle with an exposure time of 0.2 s per frame. The crystal-to-detector distance was set to 250 mm. The complete diffraction datasets were subsequently processed using *HKL-2000*^24^ and programs in *CCP4* package^25^. To capture an open state of UbiG, we prepared UbiG-phosphatidylglycerol (PG) complex by mixing 16 mg/ml protein with PG in a molecular ratio of 1:3 ∼ 1:10. Crystallization screens were performed with a Mosquito liquid-handling robot (TTP LabTech) using the vapour-diffusion method in 96-well crystallization plates at 289 K. We also tried to screen UbiG for other crystal morphologies as an alternative. However, both of these attempts were failed.

### Structure determination and refinement

The complex structure of the UbiG∆^165–187^-SAH was solved using the molecular replacement method in *Molrep*^26^, using the structure of the full-length UbiG from *E. coli* K12 (PDB code 4KDC) as the search model. The model was refined at 2.10 Å resolution using *Refmac5*^27^ and *COOT*^28^ by manual model correction. The structure factors refinement were converged to an R-factor of 17.63% and R-free of 21.52%. These final models were both evaluated with the programs *MOLPROBITY*^29^ and *PROCHECK*^30^. The data collection and structure refinement statistics were listed in Table 1. All structure figures were created using the program *PyMol* (DeLano Scientific LLC).[Table t2]

### Liposome preparation

The total lipid extract of *E. coli* (Avanti Polar Lipids, Inc) was used to generate liposomes that mimic the component of the *E. coli* plasma membrane. For liposome preparation, the total lipid extract were dissolved in chloroform in a glass tube and then was evaporated under a stream of nitrogen for 20 minutes. Next, the lipid films were dried with a vacuum pump overnight and then were hydrated at room temperature with constant mixing in buffer (20 mM Tris-HCl, 50 mM NaCl, pH 7.5). After hydration, lipid vesicles were subjected to freeze-thaw cycles in liquid nitrogen and a room temperature water bath, and then sized using Mini-Extruder Set (Avanti) with 100 nm polycarbonate filters.

### Isothermal titration calorimetry (ITC) experiments

The ITC binding studies were performed using an ITC200 (GE) at room temperature with 0.04 ml of 1 mM SAH in the injector cell and 0.26 ml of 2 mg/mL (75 mM) UbiG, UbiG mutants and liposome-bound UbiG in the sample cell, respectively. The protein and ligands were kept in a buffer consisting of 20 mM Tris-HCl (PH 7.5) and 50 mM NaCl. Five group experiments were conducted: for the first four groups, proteins (wt-UbiG and three UbiG mutants, respectively) were titrated with SAH directly, and for another group, wt-UbiG was titrated after the incubation with liposome. For the preparation of UbiG and liposome complex, 400 μg liposome was incubated with UbiG at 4 °C for 30 min. Twenty microliters injection volumes were used for all experiments. Two consecutive injections were separated by 2 min to reset the baseline. The control experiment, consisting of titration of SAH against buffer, was performed and substracted from each experiment to adjust for the heat of dilution of ligands. ITC data was analyzed with a single-site fitting model, using Origin 8.6 (OriginLab Corp).

### Analysis of methyl donor entrance

The software of *CAVER* was used to explore the putative cofactor access tunnel of UbiG. The position of SAH in the interior pocket was specified to identify tunnels directly connecting the cofactor binding site to the surface. The tunnel profile, which was the average tunnel cross-section radius along the length, was calculated from the detected accessible path.

## Additional Information

**Data availability**: The coordinates and structure factors of UbiGΔ 165–187-SAH complex were deposited in the Protein Data Bank with the access code 5DPM.

**How to cite this article**: Zhu, Y. *et al.* Structural insights into the methyl donor recognition model of a novel membrane-binding protein UbiG. *Sci. Rep.* **6**, 23147; doi: 10.1038/srep23147 (2016).

## Figures and Tables

**Table 1:** Data collection and Refinement Statistics for UbiG∆^165–187^ in complex with SAH.

Data collection statistics	UbiG∆^165–187^-SAH
Space Group	C2
Unit Cell Parameters	
*a*, *b*, *c* (Å)	139.8, 39.3, 40.1
*α*, *β*, *γ* (˚)	90.0, 94.3, 90.0◻
Wavelength (Å)	0.9792
^A^Resolution limits (Å)	50.00 − 2.10 (2.18 − 2.10)
No. of unique reflections	12887
Completeness (%)	99.2 (99.2)
Redundancy	3.5 (3.3)
B*R*_merge_ (%)	14.4 (63.3)
*R*_p.i.m_ (%)	9.0 (41.1)
Mean I/σ (I)	11.9 (3.0)
Refinement Statistics	
Resolution limits (Å)	50.00–2.10
C*R*_work_(%)/D*R*_free_(%)	17.63/21.52
Rmsd for bonds (Å)	0.008
Rmsd for angles (˚)	1.095
B factor (Å^2^)	22.73
Protein	30.04
Water	
SAH	20.70
No. of non-hydrogen protein atoms	1552
No. of water oxygen atoms	86
Ramachandran plot (%)	
most favored regions	91.9
additional allowed regions	8.1
generously allowed regions	0.0
PDB entry	5DPM

^A^Values in parentheses are for the highest resolution shell.

^B^*R*_merge _= Σh Σl |Ihl – <Ih>|/ Σh Σl <Ih>, where Ihl is the lth observation of reflection h and <Ih> is the weighted average intensity for all observations l of reflection h.

^C^*R*_work_ factor = Σh||Fobs(h)| – |Fcal(h)||/Σh|Fobs(h)|, where Fobs(h) and Fcal(h) are the observed and calculated structure factors for reflection h respectively.

^D^*R*_free_ factor was calculated same as *R*_work_ factor using the 5% the reflections selected randomly and omitted from refinement.

**Table 2 t2:** The thermodynamic parameters of the ITC experiments.

Proteins	*K*_D1_	*K*_D2_	*K*_D3_	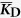	Standard Deviation
	μM	μM	μM	μM	
wt-UbiG	101.18 ± 11.17	103.91 ± 18.80	108.20 ± 21.67	104.43 ± 17.21	2.89
wt-UbiG with liposome	9.14 ± 1.63	9.09 ± 2.06	10.65 ± 2.59	9.63 ± 2.10	0.72
UbiG-M1	3.29 ± 1.10	3.48 ± 0.69	3.34 ± 0.73	3.37 ± 0.84	0.08
UbiG-M2	2.47 ± 0.19	3.27 ± 0.59	2.56 ± 0.30	2.77 ± 0.36	0.36
UbiG∆^165–187^	1.73 ± 0.24	1.73 ± 0.09	2.05 ± 0.13	1.84 ± 0.16	0.15
